# Anxiety and depression in two indigenous communities in Bangladesh

**DOI:** 10.1017/gmh.2021.33

**Published:** 2021-08-31

**Authors:** Md. Omar Faruk, Rehnuma Pervin Nijhum, Mosammat N. Khatun, Graham E. Powell

**Affiliations:** Department of Clinical Psychology, University of Dhaka, Bangladesh

**Keywords:** Anxiety, Chakma, depression, indigenous population, Marma, mental health

## Abstract

**Background:**

The mental health status of indigenous people in Bangladesh has attracted little or no attention. The objective of the present study is to determine the extent of symptoms of anxiety and depression in the two largest indigenous communities in Bangladesh.

**Methods:**

In total, 240 participants were recruited, 120 from each of the Marma and Chakma communities with an overall mean age of 44.09 years (s.d. 15.73). Marma people were older (mean ages 48.92 *v*. 39.25, *p* < 0.001). Participants completed the Anxiety Scale (AS) and Depression Scale (DS) that have been developed and standardised in Bangladesh in the Bangla (Bengali) language.

**Results:**

Results indicated that anxiety and depression scores were elevated in both communities, 59.2% of the participants scoring above the cut-off for clinical significance on AS and 58.8% of the participants scoring above the cut-off for clinical significance on DS. Marma people compared to Chakma people were more anxious (*M* = 59.49 *v*. 43.00, *p* < 0.001) and more depressed (*M* = 106.78 *v*. 82.30, *p* < 0.001). The demographic variables of age, sex and socioeconomic status were weakly or inconsistently related to scores. In the Marma people, females scored higher on both AS and DS, but in the Chakma community, males scored higher on AS and the same on DS.

**Conclusion:**

The finding of significant anxiety and depression in communities with such limited mental health services is a matter of concern and emphasises the need to formulate and implement appropriate mental health policies for indigenous people in Bangladesh and other parts of the world.

## Introduction

The issue of mental health in the world's more than 370 million indigenous people has certainly been raised as a serious matter, especially when there is a degree of international awareness, for example, in aboriginal people in Australia (Brown *et al*., 2012) or in Canada (Bellamy and Hardy, 2015*a*, *b*), but research into this issue is generally very limited to date, especially in comparison to the research into non-indigenous people (King *et al*., 2009). One of the most fundamental issues to address is simply the extent of mental health problems in indigenous peoples, because without such basic epidemiological information relating to unmet needs for treatment (Kisely *et al*., 2017), appropriate services cannot be planned or delivered. Knowledge of the mental health of indigenous people in Bangladesh is particularly lacking; therefore, in this context, the present study investigated the presence of anxiety and depression in the two largest indigenous communities in Bangladesh, the Chakma and Marma.

Worldwide, there is a reason to believe that indigenous people are particularly vulnerable to mental health issues. Around them there is rapid cultural change, they become marginalised, they can be left with little autonomy, and identity is challenged (Kirmayer *et al*., 2000). There can be childhood adversity and trauma, perceived discrimination as they develop, poverty, unemployment, sub-standard housing, food insecurity, social exclusion (Boksa *et al*., 2015). This is coupled with the exacerbating effects of poor physical health, a heightened prevalence of psychological distress being in part attributable to increased physical morbidity and disability (McNamara *et al*., 2018). Indigenous people have shorter life expectancies related to unhealthy living conditions (Durey and Thompson, 2012; Waterworth *et al*., 2015) and higher rates of chronic diseases (Conway *et al*., 2017). Indigenous people also engage more in health risk behaviour than do their non-indigenous counterparts (Waterworth *et al*., 2015).

As for the impact of these factors upon mental health, the research findings to date indicate that indigenous people do encounter a disproportionate burden of mental illness, and do experience high rates of mental health problems (Nelson and Wilson, 2017). For example, the rates of depression are higher in both male and female aboriginal peoples in Canada compared to the general population, whether residing on or off-reserve (Brown *et al*., 2012; Boksa *et al*., 2015; Bellamy and Hardy, 2015*a*, *b*). Mood-related problems, anxiety, substance use and other forms of mental disorder are more prevalent among indigenous people in Australia than non-indigenous people, whether living in remote or cosmopolitan areas (Nasir *et al*., 2018). Globally, in a meta-analysis of studies across 30 countries, indigenous people are at an increased risk of suicide compared to non-indigenous people (Pollock *et al*., 2018), and along with this risk of suicidal thoughts and attempts (Hop Wo *et al*., 2020) goes a higher risk of illicit and prescription drug use and abuse in younger indigenous people, such that the issue of suicide has been termed a youth epidemic (Leenaars, 2006).

Bangladesh is home to many indigenous communities. Estimates for the number of such communities vary because of issues in defining a community but broadly speaking estimates vary from the 27 ethnic population groups referred to in the census of 2011 to the 59 indigenous peoples listed in Roy (2012). Estimates for their total population also differ because of the problems in definition and difficulties obtaining population data, but such communities probably make up no more than 1–2% of the total population. The majority of indigenous people live in the hill tract areas in the southeastern region of Bangladesh and consist of at least 11 formally recognised indigenous ethnic groups. The Chakma and Marma tribes together constitute the majority of the indigenous population in the region and nationally are the country's two largest indigenous groups (precise Chakma and Marma population figures for the region are difficult to ascertain even given the Bangladesh Bureau of Statistics’ National Census of 2011, but are thought to be upwards of 400 000 and 200 000, respectively). The Chattogram (Chittagong) Hill Tracts (CHT) comprise three hilly districts bordering Indian states such as Tripura and Mizoram to their north and east, and Myanmar (previously Burma) to their south and east. Indigenous people in the CHT have markedly distinct customs and practices to those living in the plain lands, including the rituals at birth, death and marriage, as well as the food habit (Chakma, 2010). Chakma people speak Chakma, a mixture of Bangla, Pali and Sanskrit. The Marma people speak a Burmese dialect called Arakanese, which is also referred to as Marma. Both Chakma and Marma people follow Buddhism (Ahsan and Chakma, 1989) but in the Marma people some follow animism (Jamil and Panday, 2008). Livelihoods in both communities include weaving cloths, making baskets and manufacturing agricultural instruments, but there is also fishing, hunting and the harvesting of forest products, together with Jhum cultivation, which is rotational, shifting farming that clears land primarily by burning (Bhattacharjee *et al*., 2020), though both people have recently shifted from Jhum to plough cultivation of rice (Adnan, 2007). Research into these indigenous people has been primarily from the political, legal, economical and historical perspectives rather than from the psychological perspective (Mozumder, 2013). The situation faced in general by indigenous people in Bangladesh has, from their perspective, been set out in national campaigns (Roy, 2012; Roy and Chakma, 2015) which emphasise their marginalisation from governance and development, and which accent human rights issues.

Health coverage, especially mental health, is largely neglected in the region due to its geographical location and also what has been said to be a political unwillingness arising from ethnic clashes between the Bangalee settlers and indigenous people of the type so often experienced in other countries, too (Boksa *et al*., 2015). Further, the mental health services that do exist do not take into account the indigenous peoples' unique cultural characteristics including language and values, such that there can be a reluctance on the part of the indigenous people to access such services (Duran and Duran, 1995).

Whilst the National Mental Health Survey of Bangladesh (2019) sets out prevalence figures for depressive and anxiety disorders at the national level (6.7% and 4.5%, respectively), the present study of the Chakma and Marma people constitutes the first attempt to assess the extent of depressive and anxiety symptoms specifically in indigenous communities in the country, in order to contribute to the understanding of their mental health status and to the planning of mental health care services in their region.

## Methods

### Participants

Chakma and Marma participants were recruited from the Rangamati and Bandarban Districts, respectively, in the CHT region. Research assistants entered villages and undertook purposive, convenient sampling (Etikan *et al*., 2016) using the native language to invite villagers to participate. This approach was well received, there being no refusals to take part in the study. There were 120 Marma and 120 Chakma participants. The only inclusion criteria were age (18 or above) and membership of the community.

There were no immediate advantages to participation in the study, e.g. no monetary compensation was given. However, participants were asked to contact the researchers if they wished to know the scores and what they should do in case of high levels of anxiety and depression. They were also informed that the data would be helpful in understanding the collective experiences of anxiety and depression. In addition, they were also left with a referral directory of available mental health services so that they could seek to access care in the light of any mental health problems perceived during and after the survey.

A total of 249 persons were approached to participate. All persons approached did agree to participate, but nine persons (3.6%) declined to give complete data. In order to preserve cooperation with the communities, these persons were not asked for an explanation. These nine persons were excluded from data analysis leaving a total sample size of 240.

Villages were chosen purposively taking into account population size, accessibility and representativeness of the District as a whole. In the District of Bandarban, the Upazila (administrative region or sub-unit) of Thanchi was selected, and within Thanchi, the village of Balipara and the adjacent seven villages were again purposively chosen. Balipara is approximately 100 km from the coast and 50 km from the border with Myanmar (all distances are as the crow flies; in practice, travelling time and accessibility will reflect the nature of the terrain and the limited transport infrastructure). In the district of Rangamati, eight villages were chosen purposefully from the hilly areas away from the capital city (Rangamati). Rangamati is 170 km north of Balipara, and about 110 km from the coast and 65 km from the land border with India.

The starting point for the recruitment of participants was the community centre or clinic or equivalent in each village. These vary considerably in what resources are offered; resources that are meant to be there are often missing or inadequate, but the centres do act as general meeting points within the villages as people pass through.

### Measures

Questionnaire measures of anxiety and depression scales were chosen for the study as worldwide they represent the most common mental health conditions. The Anxiety Scale and Depression Scale used (AS and DS) were developed in Bangladesh taking Bangladeshi culture into consideration. Although Chakma and Marma communities have their own native language, the people are at least bi-lingual in the national language of Bangla. The scales were therefore presented orally or in written form in Bangla. Permissions and the demographic data were collected in the native language. Gender was male, female and third category, but third category was never used. Queries were answered and explanations were given either in Bangla or the native language as appropriate.

AS and DS were developed in Bangladesh for use in Bangladesh and so are not well known outside of this country. In order to facilitate comparison between this study and studies using other questionnaires in the West, it is appropriate to note certain psychometric properties of the scales as set out in the next sections.

We acknowledge that it is a long way preferable to have and to use tools developed or adapted to the specifically targeted population, especially an indigenous population. Sadly no such tools have been developed for use with indigenous cultures in Bangladesh, which in itself may say something about how indigenous peoples have been side-lined. Therefore, in the absence of specific tools, we had to use the scales developed for Bangalees living in plain lands as a starting point. However, before the main study, we conducted a pilot with a sample of 40 indigenous people both from Bandarban and Rangamati to check that the items were understandable and therefore potentially meaningful. As a result of this pilot, we considered that we did not need to make any *ad hoc* changes to item wording, and we were prepared for queries and questions that might arise from participants in the main study.

#### Anxiety Scale

The AS (Deeba and Begum, 2004) was developed for the Bangladeshi population. It is a 36-item questionnaire with each item rated on a five-point Likert scale, scored from 0 to 4 (‘*Never Occurs*’, ‘*Mildly Occurs*’, ‘*Moderately Occurs*’, ‘*Severely Occurs*’, ‘*Profoundly Occurs*’). Scores therefore range from 0 to 144, and scores of 48 or more are taken as being above the cut-off for clinical significance. This scale has been used extensively to measure the symptoms of anxiety in a large number of studies in Bangladesh (Nahar, 2016; Rebeiro *et al*., 2019).

Regarding AS, the mean score of non-clinical participants is 31.0 (s.d. 20.6), and for clinical participants, it is 66.0 (s.d. 19.2). Hence the cut-off of 48 or more is at approximately the 80th percentile of the non-clinical population, and approximately 83% of the clinical population scored above the cut-off. The mean score of the clinical population on AS is at approximately the 95th percentile of the non-clinical population.

#### Depression Scale

The DS (Uddin and Rahman, 2005) was also developed in Bangladesh. It is a 30-item questionnaire with each item rated on the same five-point Likert scale as the AS, but this time scored 1–5, therefore the scores range from 30 to 150. Scores of 94 or more are taken as being above the cut-off for clinical significance. This scale has also been used extensively in a large number of studies in Bangladesh (Ahmed *et al*., 2014; Gaffar and Uddin, 2016).

Regarding DS, the mean score of non-clinical participants is 77.3 (s.d. 20.5), and for clinical participants, it is 110.4 (s.d. 17.9). Hence the cut-off of 94 or more is at approximately the 80th percentile of the non-clinical population, and approximately 82% of the clinical population score above the cut-off. The mean score of the clinical population on DS is at approximately the 95th percentile of the non-clinical population.

### Procedure

The data were collected in the remote hilly areas of CHT in the Districts of Bandarban and Rangamati, in the southeastern region of Bangladesh, by four research assistants capable of speaking both Bangla and the native languages of the two communities. Explanations were given verbally in the native language and an informed consent form was obtained from each participant, a thumbprint used for those participants with no educational background.

Potential participants received an informed consent sheet on which the nature and purpose of the study was set out. They also completed a demographic information sheet. In view of limited literacy skills, the research assistants assisted as needed. The researchers and research assistants did not employ any formal script beyond this, but the research assistants had undergone appropriate training in advance to anticipate questions that might arise (training also included an understanding of the mental health problems being addressed by the study, data collection procedures and interviewing skills).

The starting point for the recruitment of participants was the community centre or clinic or equivalent in each village. These vary considerably in what resources are offered; resources that are meant to be there are often missing or inadequate, but the centres do act as general meeting points within the villages as people pass through.

It is relevant to note that the research assistants belonged to the same indigenous communities that they collected data from and spoke the same language as the participants they recruited. It would have been inappropriate to have had a Chakma person collecting data from a Marma person or *vice versa*. The work of the research assistants was voluntary, unpaid. Their subsistence expenses such as meals were covered. For the Marma community, one research assistant was an undergraduate student of Dhaka University and the other was an undergraduate student at Bandarban Government College. Both belonged to the Marma community and were recruited from the District from where the data were collected. For the Chakma community, the two research assistants were both Masters students of Clinical Psychology at Dhaka University. One was Chakma and the other, while not Chakma by direct descent was Chakma speaking and integrated into the community. All four research assistants received the same training prior to data collection.

The study followed the ethical considerations required of research involving human participants and was approved by the ethics committee of the Department of Clinical Psychology, University of Dhaka. The study ran from 2017 with all data being collected prior to the Covid-19 pandemic, so no coronavirus health precautions were required.

## Results

All data were analysed using PASW 18 (formerly SPSS).

### Demographics

The mean age of the overall sample was 44.09 years (s.d. 15.73). Mean age for the Marma people was 48.92 years (s.d. 17.32) with a range of 18 through 83 years, and for the Chakma people, mean age was 39.25 years (s.d. 12.24) with a range of 18 through 72 years. The mean age of the Marma people was significantly higher than that of the Chakma people (*t*_df 238_ = 5.00, *p* < 0.0001). The sociodemographic properties of the participants are presented in Table 1, and in this respect, the two communities did not differ; there was an approximately equal sex ratio, the vast majority of participants were married, the majority of participants were of lower or lower middle socioeconomic status (SES).

**Table 1:** Sociodemographic characteristics of the participants

Participant characteristics	Marma people		Chakma people	
	*n*	*%*	*n*	*%*
Gender of participants				
Male	67	55.8%	65	54.2%
Female	53	44.2%	55	45.8%
Socioeconomic status (SES)				
Lower	19	15.8%	21	17.5%
Lower middle	72	60.0%	91	75.8%
Middle	29	24.2%	6	5.0%
Higher	0	0.0%	2	1.7%
Marital status				
Married	111	92.5%	108	90.0%
Unmarried	5	4.2%	11	9.2%
Widow	4	3.3%	1	0.8%

In relation to marital status, it is relevant to note that in the indigenous communities that were the subject of this study, marriage is the norm, and from an early age. In the Chakma community, the average age of marriage of females is 16–20 years, and for males, it is 20–24 years. The minimum age of marriage is 16 years for girls and 20 years for boys (Chakma, 2019). The mean age of participants was well above these figures and hence the participants were long since married.

In relation to the low SES, it is relevant to note that of the overall sample, 4.2% had no literacy whatsoever (eight were Marma and two were Chakma), and for them, the questionnaires were presented entirely orally. The remaining 95.8% had varying degrees of literacy and so the research assistants assisted as necessary. There is no evidence as far as the researchers are aware that Marmas and Chakmas have differential levels of literacy, and it is worthwhile reiterating that the questionnaires were developed in Bangladesh for Bangladesh so have already been designed with weak literacy in mind.

### Relationship between demographics and scores on AS and DS

Considering the relationships between study variables in the sample as a whole (Table 2), age was weakly but significantly correlated with SES (*r* = 0.168, *p* < 0.05) and scores on DS (*r* = 0.144, *p* < 0.05). Anxiety was not correlated with demographic variables. Scores on AS and DS were significantly associated (*r* = 0.662, *p* < 0.001).

**Table 2:** Correlations between demographics and AS and DS

	Variable	Age	Gender	SES	AS	DS
1	Age	–				
2	Gender^a^	0.014	–			
3	SES^b^	0.168*	−0.006	–		
4	Anxiety	0.105	0.036	0.052	–	
5	Depression	0.144*	0.030	0.079	0.662**	–

* Correlation significant at 0.05 level.

** Correlation significant at 0.001 level.

a Point bi-serial correlation (*r*_*pb*_), male = 1, female = 2.

b Spearman rank-order correlation rho (*ρ*).

Considering the two communities separately, gender was significantly correlated with AS and DS in the Marma community (*r*s of 0.278 and 0.264, respectively, both *p* < 0.01) but not in the Chakma community. In neither community taken individually did age or SES correlate with AS or DS; therefore, the finding of an overall correlation between age and DS was due to the Marma people being both older and higher on DS rather than there being an intrinsic association between age and DS.

Overall, then, the demographic variables are only weakly related to scores on AS and DS.

### Scores of the indigenous groups on AS and DS

Considering the sample as a whole, 59.2% of the participants scored above the cut-off for clinical significance on AS and 58.8% of the participants scored above the cut-off for clinical significance on DS. Considering the two indigenous groups separately, scores for both groups on both scales were elevated. On AS, 75% of Marma and 43.33% of Chakma were above cut-off, and on DS, 82.5% of Marma and 35% of Chakma were above cut-off.

Independent samples *t* tests were performed comparing the mean scores of the two Indigenous communities on AS and DS (Table 3). Marma people scored higher on AS than Chakma people (means of 59.49 *v*. 43.00, *p* < 0.001). Similarly, scores on DS were higher among Marma people (means of 106.78 *v*. 82.30, *p* < 0.001). In both cases, a large effect size is indicated.

**Table 3:** Comparison of mean scores of indigenous groups on AS and DS

	Marma people		Chakma people		*t*	df	*p*
	*M*	s.d.	*M*	s.d.			
Anxiety	59.49	19.9	43.00	20.94	6.25	237.38	0.001
Depression	106.78	14.31	82.30	23.44	9.76	196.91	0.001

In view of the correlation between gender and AS in the Marma community noted above, independent samples *t* tests were performed comparing the mean scores of the two sexes both for the overall sample and for the two communities taken separately (Table 4).

**Table 4:** Comparison of mean scores of males and females on AS and DS

	Males		Females		*t*	df	*p*
	*M*	s.d.	*M*	s.d.			
Total sample							
AS	50.48	17.85	52.11	26.29	0.568	182.12	0.57 *n.s.*
DS	93.73	21.18	95.30	24.97	0.524	210.58	0.60 *n.s*.
Marma people							
AS	54.55	13.38	65.68	24.75	3.13	75.93	0.002
DS	103.21	13.97	111.00	13.72	3.04	111.35	0.003
Chakma people							
AS	46.35	20.77	39.04	20.63	1.93	114.97	0.05
DS	84.11	22.93	80.16	24.06	0.92	112.72	0.36 *n.s.*

There is no sex difference on AS or DS for the sample as a whole. However, in the Marma people, the females are significantly higher on both AS and DS. This sex difference was not evident in the Chakma people. In fact, in the Chakma people, the direction of the sex difference was reversed to the extent that it was Chakma males that were higher on AS.

### Variable interaction

Finally, we undertook a confirmatory two-way MANOVA with Depression and Anxiety as dependent variables and Ethnicity and Gender as fixed factors. The main effect of Ethnicity on Depression and Anxiety was significant, *F*_(2, 235)_ = 50.783, *p* = 0.000; Wilks' *λ* = 0.698 but the main effect of Gender on the dependent variables was not statistically significant, *F*_(2, 235)_ = 0.333, *p* = 0.717; Wilks' *λ* = 0.997. There was a statistically significant interaction effect between Ethnicity and Gender on the combined dependent variables, *F*_(2, 235)_ = 6.327, *p* = 0.002; Wilks' *λ* = 0.949. This confirmed that the effect of Gender across the dependent variables (Depression and Anxiety) was not the same for two different ethnicities (Chakma and Marma).

## Discussion

This study adds to the growing body of literature indicating that indigenous peoples are disproportionately burdened with mental health issues. Questionnaire measures of the symptoms of anxiety and depression were administered to a sample of 240 participants from two indigenous communities, the Marma and Chakma people, in the remote hill tract regions of southeast Bangladesh. Whilst questionnaire measures do not constitute the formal diagnosis of disorder, the scales used have been developed and standardised in Bangladesh and 59.2% of the participants scored above the cut-off for clinically significant anxiety symptoms and 58.8% scored above the cut-off for clinically significant depressive symptoms. These figures resonate with findings from around the world (Adermann and Campbell, 2007; Butler *et al*., 2007; Bellamy and Hardy, 2015*a*, *b*; Nelson and Wilson, 2017; Nasir *et al*., 2018; Balaratnasingam and Janca, 2019).

The extent of mental health issues in indigenous communities and the extent to which mental illness indicators are a real concern (referred to by Cianconi *et al*., 2019, as ‘alarming’) are to be contrasted with the limited mental health resources available to such communities. There has to be a concern that there has been a failure to recognise the needs of such communities or that known needs have been ignored, but one also has to acknowledge the challenge faced by governments such as those in Bangladesh where there are only 220 psychiatrists and 50 clinical psychologists for a population of 163 million (Alam *et al*., 2020), and indigenous people are primarily found in remote areas with a weak communications and transport infrastructure. The situation in Bangladesh has been made even more challenging for the Government by the arrival just south of the Marma and Chakma people of nearly a million Rohingya refugees from Myanmar since 2017 with their own health needs to be addressed (Government of Bangladesh and UN Refugee Agency, 2021), and who probably exhibit some of the same specific psychological needs as the indigenous people, such as the elevated risk of PTSD (Kisely *et al*., 2017).

The question has to be asked why symptoms of anxiety and depression are so prominent in the Marma and Chakma communities. Aetiology is likely to be multi-factorial, comprising stressors that are likely to be generic to indigenous populations in general, the literature citing socio-economic deprivation, educational disadvantage, unemployment, trauma, cultural disordering, loss of important ancient spiritual beliefs, exposure to pollution, market operations extracting natural resources, criminal groups, conflict and illegal exploitation of land resources, to name but a few (Uddin, 2008, 2016; Kisely *et al*., 2017; Cianconi *et al*., 2019). Another factor is likely to be that symptoms have gone untreated because of the unavailability of mental health services. In Bangladesh, for example, there is not only the shortage of professionals alluded to above but also the fact that there is only one institute of mental health and only one psychiatric hospital, located in Dhaka and Pabna, respectively, which are north of the region, well over 250 km away from the hill tract areas. This leaves indigenous people without reasonable access to either initial mental health assessment or follow-up care in the community, and gives rise to the challenge of involving traditional healers, ‘village doctors’ and other health care providers in the management of mental well-being (Alam *et al*., 2020).

Customs and practices can influence the indigenous person's understanding and explanation of psychopathology and this will in turn influence whether help is sought and if so the nature of the help sought. Further, the nature and appropriateness of the help sought will relate to how well psychopathology is managed and hence to the prevalence of active psychopathology, because without appropriate treatment, pathologies are likely to persist for longer periods. Marma people believe a supernatural power influences their birth, death and all activities in life, which has led to the performance of a range of rites and rituals (Marma, The Banglapedia, not dated). Chakmas also believe that the sacrifice of animals such as goats, chickens or ducks can calm the spirits responsible for fevers and diseases (Chakmas – Introduction, location, language, folklore, religion, major holidays, rites of passage, not dated). Mental health literacy, indeed the very concepts of mental health and mental health care, is largely absent among indigenous communities, the informal observation being that mental health symptoms are reported as somatic problems and treated as somatic issues all being treated via a spirit world. There is a need for phenomenological studies of how psychological symptoms are experienced by indigenous persons, how they are experienced and described and how they are understood in the aetiological term. It is certainly possible that differing practices between the Chakma and Marma people can lead to differentially effective management and therefore differing prevalence rates, but this possibility has to be explored in further studies.

Although custom and practice may be one factor behind the disturbing mental health figures, one has to return to the issue of historical and ongoing trauma experienced by indigenous peoples. It has been found across the globe that indigenous people can be particularly vulnerable to mental health problems (Grover, 2011). For example, Jorm *et al*. (2012) describe how indigenous Australians have a higher rate of mental disorders than does the general Australian population; 31% of Aboriginal people and 23% of Torres Strait Islanders over 18 years of age reported markedly high levels of psychological distress. But a further commonality above and beyond dry statistics are the powerful stories of trauma that these communities have to tell. Grover (2011) points out how political, economic and cultural structures can intersect in particularly harmful ways with mental health problems in indigenous communities. We ourselves did not take oral histories, we ourselves cannot offer material evidence related to past events and experiences, but the historical context relating to the Chakma and Marma people is set out by Uddin (2008) as follows. In brief, deep-rooted dissatisfaction and disaffection arose from colonisation and oppression during the British period and then the Bangalee hegemony and domination subsequent to the emergence of Bangladesh as an independent nation in 1971. Indigenous people in the CHT continued to experience oppression, marginalisation, discrimination and violence, and militarisation of the CHT resulted in restricted movement and autonomy leading to an ‘insurgency’ among indigenous people. The counter-insurgency produced cases of extra-judicial killings, torture, abduction, forced religious conversion, religious persecution, forced eviction, rape, harassment, destruction of homes and properties, and widespread arrests and detentions. It seems self-evident that there are likely to be mental health repercussions in communities subject to such experiences.

The finding in this study that symptoms of anxiety and depression are higher in the Marma people than the Chakma people is a reminder that each indigenous community has its own story, stressors and prevailing circumstances. One factor in the differential prevalence of symptoms is perhaps the extent of services available to the two communities. Chakmas have been said to have more opportunities and facilities in general compared to other ethnic groups (Roy *et al*., 2008). It is generally difficult in Bangladesh to obtain up to date and detailed information about specific health-related services, but Masud Ahmed (2001) found that Chakma and Bangalee villages had more treatment facilities and Marma villages had a smaller number of doctors and para-professionals compared to other ethnic groups. In the hill tracts, better facilities are associated with more health-seeking behaviour (Masud Ahmed, 2001), and in general, in other parts of the world, too, proximity to health facilities in resource-poor settings increases the likelihood of health care seeking (Anselmi *et al*., 2015). It seems reasonable to suppose that the provision of health facilities is likely to be beneficial to the health of the community, but this will be only one factor and further research is needed into the community-specific factors and stressors that relate to mental health.

With regard to the issue of community specificity, the Districts of Bandarban and Rangamati were purposefully selected for this study because Marmas and Chakmas, the two largest indigenous populations, predominantly live in these two districts. We acknowledge that Tripura is the third largest indigenous community based in the District of Khagracchari. We hope that the reader will understand that any study has limited resources and that our inability to extend the study to a third indigenous group does not imply any disrespect towards the Tripura community or in any way reflect a downplaying of problems they are experiencing. Indeed, it is a premise of our study that all indigenous communities should be able to access such mental health services as are required.

Finally, there is the intriguing finding that while females in the Marma people are higher on symptoms of anxiety and depression, which is the typical finding worldwide (Breslau *et al*., 1995; Altemus, 2006; Altemus *et al*., 2014), this trend is reversed in the Chakma people, significantly so in relation to anxiety symptoms, on which Chakma males score higher. We have no explanation for this, but it is a reminder that sex differences are not necessarily biologically driven. It will be important in future studies to look at gender-specific factors and stressors within each community taken separately.

An exploratory study such as this clearly raises many questions and opens many avenues for further research. There needs to be a full epidemiological study of mental health in the indigenous peoples of Bangladesh equipped to make formal diagnoses of not just affective disorders but the full range of mental health issues including neurodevelopmental disorder, psychosis, trauma-related disorders and substance-related and addictive disorders. There needs to be a better understanding of perceived pressures and stressors in such communities. There needs to be a better understanding of how they perceive and conceptualise mental illness, and how they might view non-traditional treatments such as medication and talking therapies.

## Conclusions

This study of the mental health status of the two largest indigenous peoples in the hill tract regions of Bangladesh highlights the need for mental health interventions in their communities and will help formulate long-term inclusive mental health strategies. For the moment, though, the most important implication of this study, while the long-term piece of work is being done, is that such health facilities as exist and such health workers as are present need to be alert to mental health issues and to such first aid mental health strategies as might be possible.
